# The impact of IgG subclass deficiency on the risk of mortality in hospitalized patients with COPD

**DOI:** 10.1186/s12931-022-02052-3

**Published:** 2022-05-31

**Authors:** Hyun Lee, Cara Kovacs, Andre Mattman, Zsuzsanna Hollander, Virginia Chen, Raymond Ng, Janice M. Leung, Don D. Sin

**Affiliations:** 1 Centre for Heart Lung Innovation, University of British Columbia, St. Paul’s Hospital, Vancouver, BC Canada; 2 Department of Internal Medicine, Hanyang University College of Medicine, Seoul, Republic of Korea; 3 Department of Pathology and Laboratory Medicine, University of British Columbia, Vancouver, BC Canada; 4 PROOF Centre of Excellence, University of British Columbia, Vancouver, BC Canada; 5 Division of Respiratory Medicine, Department of Medicine, University of British Columbia, Vancouver, BC Canada

**Keywords:** IgG, IgG subclass deficiency, COPD, Mortality

## Abstract

**Background:**

Immunoglobulin G (IgG) deficiency increases the risk of acute exacerbations and mortality in chronic obstructive pulmonary disease (COPD). However, the impact of IgG subclass deficiency on mortality in COPD is unknown. Here, we determined which IgG subclass, if any, is associated with increased risk of mortality in COPD.

**Methods:**

We measured serum IgG subclass concentrations of 489 hospitalized patients with COPD who were enrolled in the Rapid Transition Program (clinicaltrials.gov identifier NCT02050022). To evaluate the impact of IgG subclass deficiency on 1-year mortality, Cox proportional hazards regression analyses were performed with adjustments for potential confounders.

**Results:**

Deficiencies in IgG1, IgG2, IgG3, and IgG4 were present in 1.8%, 12.1%, 4.3%, and 11.2% of patients, respectively. One-year mortality was 56% in patients with IgG1 deficiency, 27% in IgG2 deficiency, 24% in IgG3 deficiency, and 31% in IgG4 deficiency. Cox proportional modeling showed that IgG1 and IgG4 deficiencies increased the 1-year mortality risk with an adjusted hazard ratio of 3.92 (95% confidence interval [CI] = 1.55–9.87) and 1.74 (95% CI = 1.02–2.98), respectively. Neither IgG2 nor IgG3 deficiency significantly increased 1-year mortality. Two or more IgG subclass deficiencies were observed in 5.3%. Patients with 2 or more IgG subclass deficiencies had a higher 1-year mortality than those without any deficiencies (46.2% vs. 19.7%, p < 0.001), with an adjusted hazard ratio of 2.22 (95% CI = 1.18–4.17).

**Conclusions:**

IgG1 and IgG4 deficiency was observed in 1.8% and 11.2% of hospitalized patients with COPD, respectively, and these deficiencies were associated with a significantly increased risk of 1-year mortality.

**Supplementary Information:**

The online version contains supplementary material available at 10.1186/s12931-022-02052-3.

## Background

Despite recent advances in the treatment of chronic obstructive pulmonary disease (COPD), it is the 3rd leading cause of death, responsible for over 3 million deaths per year worldwide [1]. Most deaths occur during periods of acute exacerbations (AECOPDs) [2], which are largely triggered by viral or bacterial respiratory tract infections [3, 4]. One important risk factor for recurrent respiratory tract infections is humoral immune deficiency caused by hypogammaglobulinemia [5].

In blood, immunoglobulin G (IgG) is the predominant circulating antibody that plays a crucial role in preventing severe respiratory tract infections [5–8]. Approximately 10–28% of patients with moderate to severe COPD demonstrate hypogammaglobulinemia, which in turn, has been associated with an increased risk for exacerbations, hospitalizations, and mortality [5, 7, 9–11]. There are four distinct subclasses of IgG (IgG1, IgG2, IgG3, and IgG4), and each has a slightly different structure and function in the immune system [12, 13]. While the relationship between total IgG levels and mortality has been established in COPD [5, 11], the impact of IgG subclass deficiencies on COPD remains obscure, especially in hospitalized patients with COPD who are at increased risk of mortality. To date, there has been a paucity of studies with sufficient size and scope that have evaluated IgG subclass deficiencies in hospitalized patients with COPD. Studies have moreover used cross-sectional methodologies, limiting causal inferences [14, 15]. Here, we describe the prevalence of IgG subclass deficiencies and their relationship with 1-year mortality in hospitalized patients with COPD.

## Materials and methods

### Study participants

**Fig. 1:**
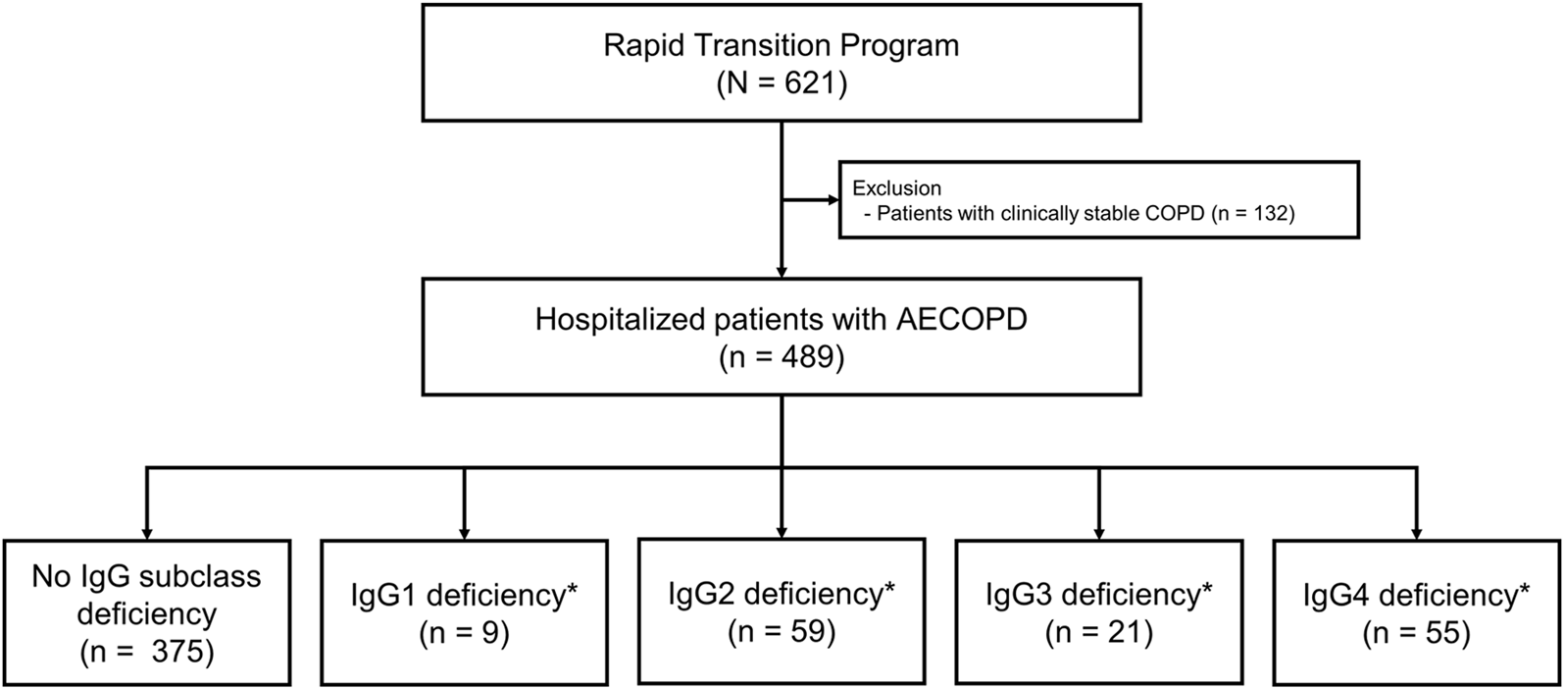
Study flow chart. *Some patients had two or more IgG subclass deficiencies. *IgG* immunoglobulin G

The Rapid Transition Program (ClinicalTrials.gov number: NCT02050022) included hospitalized patients with AECOPD (n = 489) and clinically stable patients with COPD (n = 132) who were seen and managed at St. Paul’s Hospital or Vancouver General Hospital, both in Vancouver, Canada between April 2013 and December 2017 (Fig. 1). The blood samples for hospitalized patients were collected on the first three days of their hospitalization.

### Ethical statement

This study was approved by the research ethics board of each institution (certificate number H11-00786 for St. Paul’s Hospital and certificate number H13-00790 for Vancouver General Hospital). Informed consent to participate in this study was received from each patient. None of the patients had bronchiectasis by history or chest imaging. Patients were followed for 1 year after enrollment. Mortality was ascertained through medical records and validated by death certificates. The detailed information about this study was previously published [16].

### Study outcome

The primary aim was to evaluate the impact of IgG subclass deficiencies on 1-year mortality in hospitalized patients with COPD.

### Measurements of IgG subclasses

Serum samples were processed using standardized protocols and stored in a -80ºC freezer and thawed once for the assay. We measured IgG subclass concentrations in serum using liquid chromatography-tandem mass spectrometry (LC–MS/MS) as previously described [17]. The processing and measurement of all samples were performed in the clinical laboratory at St. Paul’s Hospital, Vancouver, British Columbia, Canada. IgG subclass deficiency was defined when serum IgG subclass levels were below their respective normal ranges (i.e., fell below 2 standard deviations [SDs] from the mean concentrations in a healthy population): IgG1 < 2.80 g/L, IgG2 < 1.15 g/L, IgG3 < 0.24 g/L, and IgG4 < 0.052 g/L [18].

### Statistical analysis

Continuous variables are presented as mean ± SD or median (interquartile range). Continuous variables were compared using a t-test or a Mann-Whitney U test, as appropriate. Categorical variables are presented as numbers (%) and were compared using a Chi-squared test or a Fisher’s exact test, as appropriate. To evaluate the survival between subjects with an IgG subclass deficiency and those with no IgG subclass deficiency, we performed a log-rank test. For the analyses of survival according to the number of IgG subclass deficiencies (0, 1, and 2 or more), Bonferroni adjustment was made to account for multiple comparisons. To evaluate the impact of IgG subclass deficiency vs. no IgG subclass deficiency on 1-year mortality, Cox proportional hazards regression analysis was performed with adjustments for age, sex, ethnicity (white vs. other ethnicities), smoking status (current vs. non-current), presence of asthma, and cardiac comorbidity status (i.e., a history of heart failure, myocardial infarction, stable coronary disease, or coronary artery bypass graft surgery). For the Cox analyses, we tested the proportional hazard assumption visually and by using Schoenfeld residuals and the assumption was met except for the IgG2 analysis. All tests were two-sided and p values < 0.05 were considered statistically significant. Data analysis was performed using R software version 4.1.0 (The R Foundation for Statistical Computing Platform, Vienna, Austria), RStudio software version 1.4.1717 (RStudio, Boston, MA), and STATA 15.1 version (StataCorp LP, College Station, TX, USA).

## Results

### Study participants

The baseline characteristics of 489 patients are summarized in Table 1. The mean (± SD) age of the study population was 67.3 ± 11.6 years and 63.2% were males. Deficiencies in IgG1, IgG2, IgG3, and IgG4 were present in 1.8% (n = 9), 12.1% (n = 59), 4.3% (n = 21), and 11.2% (n = 55) of the cohort, respectively. Approximately 5% of the patients (n = 26) demonstrated two or more IgG subclass deficiencies. The baseline characteristics of patients with each IgG subclass deficiency are summarized in Additional file 1: Table S1.

**Table 1:** Baseline characteristics of study participants

	Total (N = 489)
Age, years	67.3 ± 11.6
Male	309 (63.2)
Ethnicity, white	391 (81.0)
Smoking status	
Current smoker	287 (58.7)
Ex-smoker	173 (35.4)
Never smoker	29 (5.9)
Asthma	119 (24.4)
Cardiac comorbidities*	182 (37.3)
Lung function	
Post-bronchodilator FVC, L	2.8 ± 1.1
Post-bronchodilator FVC, %predicted	77.6 ± 23.1
Post-bronchodilator FEV_1_, L	1.5 ± 0.8
Post-bronchodilator FEV_1_, %predicted	53.7 ± 23.8
Post-bronchodilator FEV_1_/FVC	45.2 ± 24.8
IgG subclass deficiency	
IgG1 deficiency	9 (1.8)
IgG2 deficiency	59 (12.1)
IgG3 deficiency	21 (4.3)
IgG4 deficiency	55 (11.3)
1-year mortality	101 (20.7)

Data are presented as numbers (%) or mean ± SD

*FVC* forced vital capacity, *FEV1* forced expiratory volume in 1 s, *IgG* immunoglobulin G

*Cardiac comorbidities included a history of heart failure, myocardial infarction, stable coronary disease, or coronary artery bypass graft surgery

### Impact of IgG subclass deficiency on 1-year mortality

**Fig. 2:**
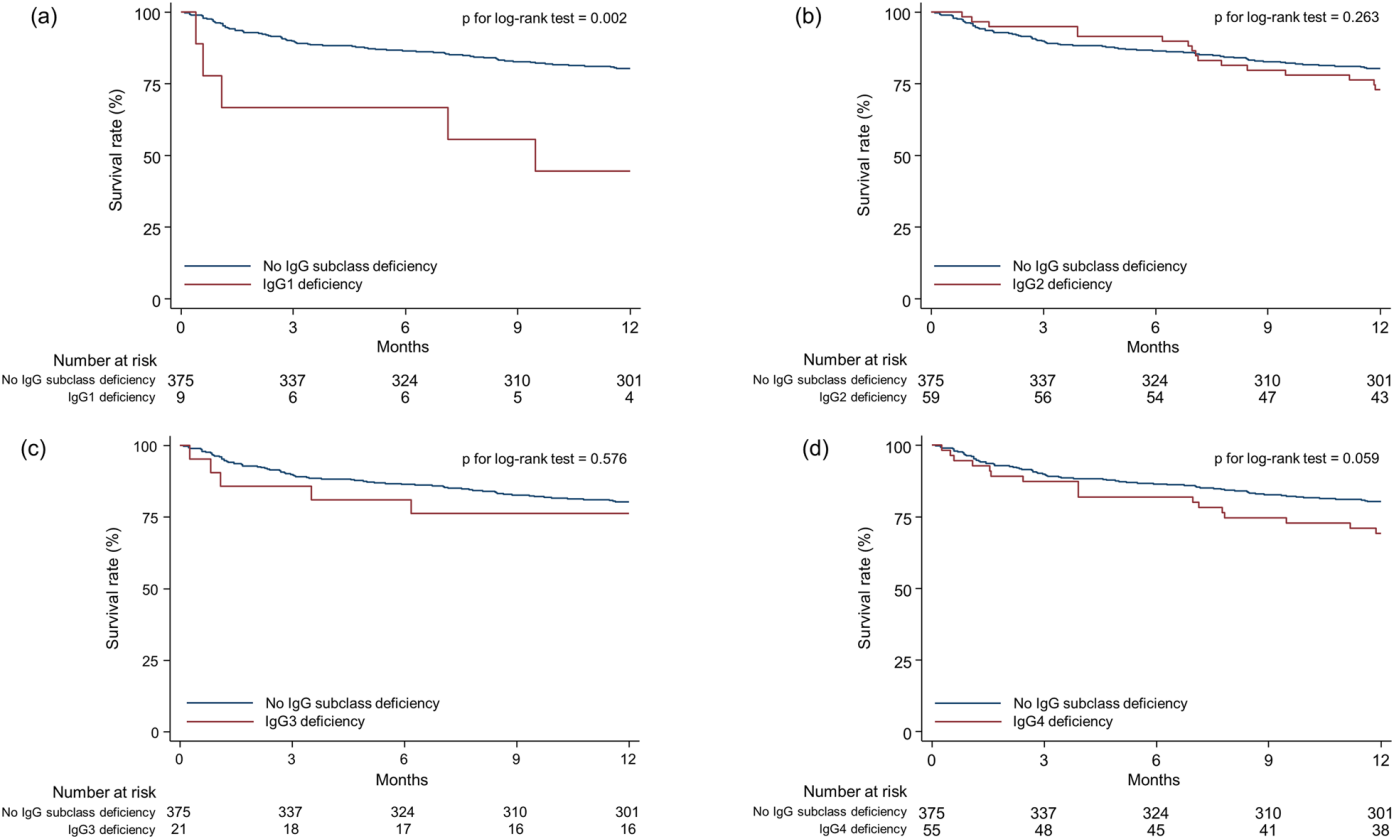
Kaplan**–**Meier survival curves for mortality according to IgG subclass deficiency (**a**) IgG1 deficiency; (**b**) IgG2 deficiency; (**c**) IgG3 deficiency; (**d**) IgG4 deficiency. *IgG* immunoglobulin G

The overall 1-year mortality rate of the study population was 20.7% (101/489): 55.6% (5/9) in patients with IgG1 deficiency, 27.1% (16/59) in IgG2 deficiency, 23.8% (5/21) in IgG3 deficiency, and 30.9% (17/55) in IgG4 deficiency, respectively. Multivariable Cox regression analyses revealed significant relationships between IgG1 and IgG4 deficiencies and increased 1-year mortality (Table 2). Patients with IgG1 deficiency were 3.92 (95% confidence interval [CI] = 1.55–9.87) times more likely to experience 1-year mortality compared to those without IgG1 deficiency. Patients with IgG4 deficiency also had a higher risk of 1-year mortality compared to those without IgG4 deficiency (adjusted hazard ratio [HR] = 1.74; 95% CI = 1.02–2.98). In contrast, IgG2 (unadjusted HR = 1.36; 95% CI = 0.79–2.33) and IgG3 deficiency (adjusted HR = 1.27; 95% CI = 0.51–3.15) were not associated with increased risk of mortality in patients with COPD. The proportional hazards assumption was not met for the analysis of IgG2 deficiency and mortality. Survival analyses showed similar results (Fig. 2a–d).

**Table 2:** Unadjusted and adjusted HRs related to 1-year mortality according to IgG subclass deficiency

Type of IgG deficiency	Number at risk	1-year mortality	Unadjusted model		Adjusted model^*^	
			Unadjusted HR (95% CI)	p value	Adjusted HR (95% CI)	p value
IgG1 deficiency	9	55.6% (5/9)	3.80 (1.54– 9.41)	0.004	3.92 (1.55–9.87)	0.004
IgG2 deficiency^†^	59	27.1% (16/59)	1.36 (0.79–2.33)	0.265	NA^†^	NA^†^
IgG3 deficiency	21	23.8% (5/21)	1.29 (0.52–3.20)	0.577	1.27 (0.51–3.15)	0.612
IgG4 deficiency	55	30.9% (17/55)	1.65 (0.97–2.79)	0.063	1.74 (1.02–2.98)	0.043

Data are presented as number, percentage, or ratios (95% CIs)

*HR* hazard ratio, *IgG* immunoglobulin G, *CI* confidence interval

*Adjusted for age, sex, ethnicity (white vs. other ethnicities), smoking status (current vs. non-current), asthma status, and cardiac comorbidity status

^†^The proportional hazards assumption was not met

Besides IgG subclass deficiencies, there were other risk factors for 1-year mortality in COPD patients. As shown in Additional file 2: Table S2, whereas age (except for IgG4 subclass deficiency) and male sex increased the risk of 1-year mortality, there were no significant associations between ethnicity, smoking status, asthma, and cardiac comorbidities and 1-year mortality.

### Impact of the number of IgG subclass deficiency on mortality

**Fig. 3:**
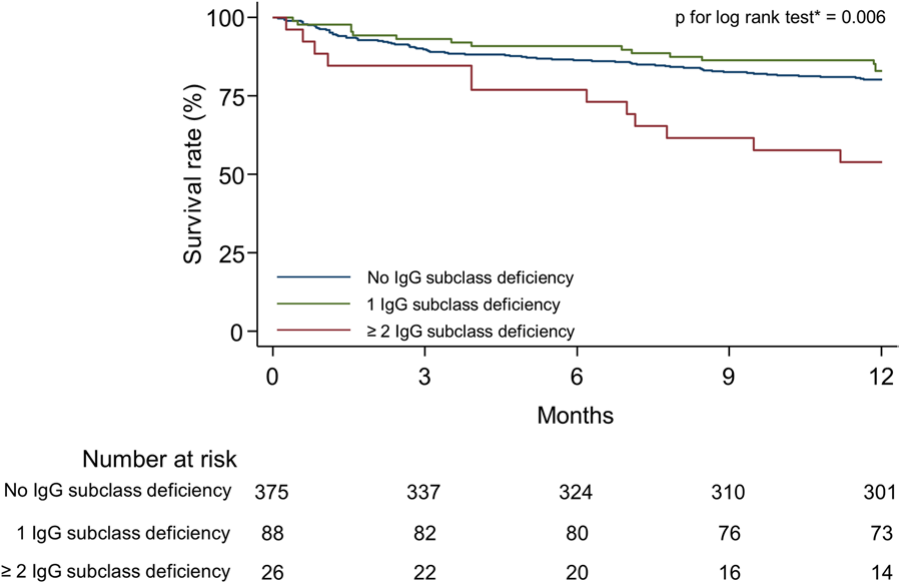
Kaplan–Meier survival curves for mortality according to the number of IgG subclass deficiencies. Bonferroni adjustment was performed for multiple comparisons. *IgG* immunoglobulin G

The 1-year mortality according to the number of IgG subclass deficiency is as follows: 19.7% [74/375] in those without any IgG subclass deficiencies, 17.1% [15/88] in those with 1 subclass deficiency, 39.1% [9/23] in those with 2 subclass deficiencies, 100% [2/2] in those with 3 deficiencies, and 100% [1/1] in one individual with deficiencies in all 4 subclasses. Patients with 2 or more deficiencies had a significantly higher 1-year mortality than those without any deficiencies or those with 1 deficiency (46.2% vs. 19.7% vs. 17.1%; p < 0.001), and there was a significant difference in the survival rate between the former and the two latter groups (p for log-rank test = 0.006) (Fig. 3). In multivariable Cox-regression analyses, patients with 2 or more deficiencies were 2.22 (95% CI = 1.18–4.17) and 2.44 (95% CI = 1.11–5.33) times more likely to experience 1-year mortality than those without any IgG subclass deficiencies or those with 1 deficiency, respectively (Table 3).

**Table 3:** Unadjusted and adjusted HRs related to 1-year mortality according to the number of IgG subclass deficiency

Number of IgG deficiency	Number at risk	1-year mortality	Unadjusted model		Adjusted model*	
			Unadjusted HR (95% CI)	p value	Adjusted HR (95% CI)	p value
0	375	19.7% (74/375)	Reference		Reference	
1	88	17.1% (15/88)	0.84 (0.48–1.46)	0.528	0.91 (0.52–1.59)	0.736
2 or more	26	46.2% (12/26)	2.70 (1.47–4.98)	0.001	2.22 (1.18–4.17)	0.014

Data are presented as numbers, percentages, or ratios (95% CIs)

*HR* hazard ratio, *IgG* immunoglobulin G, *CI* confidence interval

*Adjusted for age, sex, ethnicity (white vs. other ethnicities), smoking status (current vs. non-current), asthma status, and cardiac comorbidity status

## Discussion

Hypogammaglobulinemia affects approximately 1 in 5 patients with moderate to severe COPD. However, the occurrence of individual IgG subclass deficiency in hospitalized COPD patients at a high risk of morbidity and mortality has not been well studied. Here, we showed that the probability of individual subclass deficiency in a high-risk group of patients was 1.8% for IgG1, 12.1% for IgG2, 4.3% for IgG3, and 11.2% for IgG4. Importantly, we found that 1-year mortality was highest in patients with IgG1 deficiency (50%), followed by IgG4 deficiency (27.1%), IgG2 deficiency (25.7%), IgG3 deficiency (19.4%), and lowest in those without any IgG subclass deficiencies (16.7%). Independent of age, sex, ethnicity, smoking status, asthma history, and cardiac comorbidities, IgG1 and IgG4 deficiency elevated the risk of mortality by 3.9-fold and 1.7-fold, respectively.

The overall occurrence of any IgG subclass deficiency in this study was 23.3%, which is similar to the findings reported previously (17.7–25.9%) [9, 15, 19]. However, the distribution of the IgG subclass deficiencies in this study was different from those of previous studies. We showed that the most common deficiencies were related to IgG2 and IgG4. In contrast, previous studies have shown IgG3 deficiency to be the most common [6, 9, 15] except for one notable study where IgG2 deficiency was the most prevalent [19]. Although the reasons for this discordance are not clear, it has been previously suggested that the distribution of IgG subclass deficiency varies according to the severity of COPD with the risk of IgG2 deficiency rising with increasing frequencies of AECOPDs [9]. The relatively high rates of IgG2 deficiency in the present study, which contained patients who were hospitalized with AECOPD, are consistent with this previous observation.

We previously showed that hypogammaglobulinemia is associated with an increased risk of mortality in patients with COPD [11]. We extend these findings by demonstrating increased 1-year mortality with IgG1 or IgG4 but not IgG2 or IgG3 subclass deficiency. The exact mechanisms by which IgG1 and IgG4 deficiencies confer increased mortality in patients with COPD are obscure and were beyond the purview of the present study. However, since most deaths occur during periods of severe lower respiratory tract infection, we posit that IgG1 deficiency increases the risk for severe AECOPDs [9]. This raises the possibility that Ig replacement therapy might improve survival in these patients. However, this hypothesis will require validation in a large randomized controlled trial before it can be implemented routinely in clinical practice.

Interestingly, we also found that IgG4 deficiency was associated with increased mortality. However, in our previous study which evaluated different cohorts of patients with stable COPD, we did not find that IgG4 deficiency was associated with increased risk of AECOPD or AECOPD-related hospitalization [9]. This raises the possibility that the impact of IgG4 deficiency on mortality may be indirect. Consistent with this notion, recent studies have shown that low serum IgG4 increases the risk of not only respiratory infections but also non-respiratory conditions including allergic, autoimmune, and cardiovascular diseases [20–26]. For these latter conditions, serum IgG levels appear to be inversely related to the severity of disease [23, 25, 26]. Thus, IgG4 deficiency might be a biomarker of increased burden of co-morbidity or may reflect the severity of the underlying COPD. Future studies should evaluate the potential causal role, if any, that IgG4 deficiency plays in the pathogenesis of COPD. We also found that the risk of mortality increases monotonically with the number of subclass deficiencies.

Unexpectedly, IgG2 deficiency, a predictor of AECOPD or hospitalization in the previous study of patients with stable COPD [9], was not associated with an increased risk of mortality. Although the reasons for this phenomenon are not clear, it should be noted that there are important differences in the severity of COPD between the current cohort and the one previously described [9]. In the current cohort, hospitalized patients with an AECOPD were enrolled; in contrast, all of the patients in the previous study [9] were stable at the time of recruitment. Furthermore, 12.1% of the patients in the present demonstrated IgG2 deficiency; whereas only 5.7% demonstrated IgG2 deficiency in the previous study.

In the era of personalized medicine, identification of Ig subclass deficient patients is attractive as it may be a modifiable and a treatable trait. Whereas total IgG deficiency is relatively common in COPD patients (~ 20%), subclass deficiency is rare. Indeed, we found that only 1.6% of our patients were IgG1 deficient and 5% were deficient in two or more subclasses. Given the cost and inconvenience of Ig replacement therapy, it may be more reasonable to target this therapy for those with subclass deficiencies rather than those with hypogammaglobulinemia based on total serum IgG levels. To date, only a few small observational studies have evaluated the possible salutary effects of replacement therapy in patients with COPD who also demonstrated hypogammaglobulinemia. One study of 14 patients showed that the administration of intravenous Ig was associated with a 90% reduction in the occurrence of AECOPD in predominantly hypogammaglobulinemic COPD patients [8]. Another study of 22 patients with hypogammaglobulinemia demonstrated that prophylactic antibiotics or Ig therapy might be beneficial in reducing the frequency of AECOPDs [27]. Because these study results were limited by study design and the small sample sizes, well-designed randomized controlled trials targeting IgG subclass deficiencies are needed to validate the promise of these early reports.

There were several limitations to the current study. First, clinically, it is generally recommended to measure IgG levels twice at least 1 month apart to determine IgG subclass deficiency [28, 29]. However, in the present study, IgG levels were measured only once at baseline. Moreover, for hospitalized patients, we were able to measure IgG levels only during the acute setting. It should be noted, however, that serum IgG levels are relatively stable and not affected by acute illness. The half-life of IgG subclasses is ~ 20–30 days [30]. Second, we could not determine the causes for the IgG subclass deficiencies. However, this was beyond the purpose of our study. Third, since this study included a small number of stable COPD patients, studies evaluating the impact of IgG subclass deficiencies on mortality in patients with stable COPD are needed. Although there was no significant association between IgG subclass deficiencies and mortality in stable COPD patients (Additional file 3: Table S3, Additional file 4: Table S4, Additional file 5: Table S5), these results are likely to be underpowered due to the small number of patients. Fourth, we did not collect data on causes of mortality as many died outside of the hospital. However, ascertaining and attributing causes to mortality are extremely challenging in COPD, who often harbor multiple co-morbidities. A previous study suggests that for hospitalized or recently hospitalized patients, respiratory tract infections, pulmonary embolism and cardiac failure are the leading causes of death [31]. How IgG subclass deficiencies modulate these terminal endpoints in COPD is uncertain. Future studies are needed to determine the mechanisms by which IgG subclass deficiencies lead to mortality in COPD patients.

## Conclusions

IgG1 deficiency, through rare (1.8%), was associated with a 3.9-fold increased risk of 1-year mortality in patients with COPD. One in 9 patients with COPD had IgG4 deficiency, which increased mortality by 1.7-fold. Our study suggests that IgG subclass measurements can be useful to classify hospitalized patients with COPD at a high risk of mortality. These patients may be targets for future Ig replacement studies.

## Supplementary Information

**Additional file 1.** Table S1. Baseline characteristics of study participants with each IgG subclass deficiency.

**Additional file 2.** Table S2. Adjusted HRs† of clinical risk factors for 1-year mortality.

**Additional file 3.** Table S3. Baseline characteristics of subjects with stable COPD.

**Additional file 4.** Table S4. Unadjusted and adjusted HRs related to 1-year mortality according to IgG subclass deficiency in stable COPD patients.

**Additional file 5.** Table S5. Unadjusted and adjusted HRs related to 1-year mortality according to the number of IgG subclass deficiency in stable COPD patients.

## Data Availability

The datasets used and/or analysed during the current study are available from the corresponding author on reasonable request.

## Abbreviations

AECOPD: Acute exacerbation of COPD
CI: Confidence interval
COPD: Chronic obstructive pulmonary disease
FEV_1_: Forced expiratory volume in 1 s
FVC: Forced vital capacity
HR: Hazard ratio
Ig: Immunoglobulin
