# Supplementary material for: The impact of IgG subclass deficiency on the risk of mortality in hospitalized patients with COPD
Source: Respir Res. 2022 May 31;23:141. doi: 10.1186/s12931-022-02052-3 (PMC9158163; doi:10.1186/s12931-022-02052-3)
Supplement: Supplementary file 1 — Additional file 1. Table S1. Baseline characteristics of study participants with each IgG subclass deficiency. [file 12931_2022_2052_MOESM1_ESM.docx]

**Table S1.** Baseline characteristics of study participants with each IgG subclass deficiency

|  | IgG1 deficiency  (n = 9) | IgG2 deficiency  (n = 59) | IgG3 deficiency  (n = 21) | IgG4 deficiency  (n = 55) |
| --- | --- | --- | --- | --- |
| Age, years | 72.6 ± 11.0 | 66.8 ± 9.8 | 70.2 ± 9.9 | 67.8 ± 12.5 |
| Male | 5 (55.6) | 35 (59.3) | 14 (66.7) | 26 (47.3) |
| Ethnicity, whites | 8 (88.9) | 51 (86.4) | 20 (95.2) | 43 (78.2) |
| Smoking status |  |  |  |  |
| Current smoker | 3 (33.3) | 37 (62.7) | 13 (61.9) | 28 (50.9) |
| Ex-smoker | 6 (66.7) | 21 (35.6) | 4 (19.1) | 24 (43.6) |
| Never smoker | 0 (0) | 1 (1.7) | 4 (19.1) | 3 (5.5) |
| Asthma | 3(33.3) | 10 (17.0) | 5 (25.0) | 14 (25.5) |
| Cardiac Comorbidities^*^ | 6 (66.7) | 29 (49.2) | 11 (55.0) | 23 (41.8) |
| Lung function |  |  |  |  |
| Post-Bronchodilator FVC, L | 1.5 ± 0.2 | 2.2 ± 0.9 | 3.2 ± 1.1 | 2.4 ± 0.9 |
| Post-Bronchodilator FVC, %predicted | 52.0 ± 11.3 | 69.6 ± 25.3 | 92.3 ± 18.9 | 76.6 ± 21.9 |
| Post-Bronchodilator FEV_1_, L | 0.8 ± 0.1 | 1.1 ± 0.6 | 1.8 ± 0.8 | 1.4 ± 0.7 |
| Post-Bronchodilator FEV_1_, %predicted | 37.0 ± 8.5 | 42.3 ± 21.1 | 69.5 ± 23.1 | 54.7 ± 22.0 |
| Post-Bronchodilator FEV_1_/FVC | 66.5 ± 4.9 | 61.5 ± 18.3 | 74.3 ± 15.3 | 64.9 ± 14.4 |
| IgG subclass level, g/L | 2.35 (2.16–2.61) | 0.80 (0.72–0.94) | 0.18 (0.12–0.20) | 0.03 (0.02–0.04) |
| 1-year mortality | 5 (55.6) | 16 (27.1) | 5 (23.8) | 17 (30.9) |

Data are presented as numbers (%), mean ± SD, or median (interquartile range).

^*^ Cardiac comorbidities included a history of heart failure, myocardial infarction, stable coronary disease, or coronary artery bypass graft surgery.

*Abbreviations*: IgG, immunoglobulin G; FVC, forced vital capacity; FEV_1_, forced expiratory volume in 1 second.
