# Supplementary material for: The impact of IgG subclass deficiency on the risk of mortality in hospitalized patients with COPD
Source: Respir Res. 2022 May 31;23:141. doi: 10.1186/s12931-022-02052-3 (PMC9158163; doi:10.1186/s12931-022-02052-3)
Supplement: Supplementary file 2 — Additional file 2. Table S2. Adjusted HRs† of clinical risk factors for 1-year mortality. [file 12931_2022_2052_MOESM2_ESM.docx]

**Table S2.** Adjusted HRs^†^ of clinical risk factors for 1-year mortality

|  | IgG1 deficiency | | IgG2 deficiency | | IgG3 deficiency | | IgG4 deficiency | |
| --- | --- | --- | --- | --- | --- | --- | --- | --- |
|  | Adjusted HR^*^  (95% CI) | p value | Adjusted HR^†^  (95% CI) | p value | Adjusted HR^*^  (95% CI) | p value | Adjusted HR^*^  (95% CI) | p value |
| IgG subclass deficiency | 3.92 (1.55–9.87) | 0.004 | NA^†^ | NA^†^ | 1.27 (0.51–3.15) | 0.612 | 1.74 (1.02–2.98) | 0.043 |
| Age | 1.02 (1.001–1.48) | 0.041 | NA^†^ | NA^†^ | 1.02 (1.001–1.05) | 0.040 | 1.02 (0.99–1.04) | 0.054 |
| Sex, male | 1.99 (1.16–3.42) | 0.012 | NA^†^ | NA^†^ | 2.12 (1.22–3.70) | 0.007 | 1.73 (1.07–2.80) | 0.025 |
| White | 0.83 (0.45–1.55) | 0.564 | NA^†^ | NA^†^ | 0.78 (0.41–1.48) | 0.443 | 0.71 (0.38–1.31) | 0.277 |
| Current smoker | 1.30 (0.79–2.12) | 0.300 | NA^†^ | NA^†^ | 1.23 (0.76-2.01) | 0.399 | 1.08 (0.68–1.72) | 0.746 |
| Asthma | 0.57 (0.30–1.06) | 0.074 | NA^†^ | NA^†^ | 0.54 (0.29–1.04) | 0.065 | 0.32 (0.31–1.07) | 0.081 |
| Cardiac comorbidities | 1.37 (0.86–2.18) | 0.186 | NA^†^ | NA^†^ | 1.31 (0.82–2.08) | 0.263 | 1.47 (0.95–2.27) | 0.082 |

Data are presented as ratios and 95% CIs.

^*^Each Cox regression model was adjusted for age, sex, ethnicity (white vs. other ethnicities), smoking status (current vs. non-current), asthma status, and cardiac comorbidity status in addition to the corresponding IgG deficiency.

^†^The proportional hazards assumption was not met for the analysis of IgG2 deficiency and mortality.

***Abbreviations*:** HR, hazard ratio; IgG, immunoglobulin G; CI, confidence interval.
