# Supplementary material for: The impact of IgG subclass deficiency on the risk of mortality in hospitalized patients with COPD
Source: Respir Res. 2022 May 31;23:141. doi: 10.1186/s12931-022-02052-3 (PMC9158163; doi:10.1186/s12931-022-02052-3)
Supplement: Supplementary file 3 — Additional file 3. Table S3. Baseline characteristics of subjects with stable COPD. [file 12931_2022_2052_MOESM3_ESM.docx]

**Table S3.** Baseline characteristics of subjects with stable COPD

|  | Total (N = 132) |
| --- | --- |
| Age, years | 65.4 ± 11.4 |
| Male | 93 (70.5) |
| Ethnicity, white | 122 (92.4) |
| Smoking status |  |
| Current smoker | 59 (44.7) |
| Ex-smoker | 64 (48.5) |
| Never smoker | 9 (6.8) |
| Asthma | 23 (17.6) |
| Cardiac comorbidities^*^ | 28 (21.4) |
| Lung function |  |
| Post-bronchodilator FVC, L | 2.9 ± 1.1 |
| Post-bronchodilator FVC, %predicted | 78.3 ± 21..2 |
| Post-bronchodilator FEV_1_, L | 1.5 ± 0.8 |
| Post-bronchodilator FEV_1_, %predicted | 50.2 ± 21.2 |
| Post-bronchodilator FEV_1_/FVC | 49.2 ± 15.2 |
| IgG subclass deficiency |  |
| IgG1 deficiency | 1 (0.8) |
| IgG2 deficiency | 11 (8.3) |
| IgG3 deficiency | 10 (7.6) |
| IgG4 deficiency | 15 (11.4) |
| 1-year mortality | 9 (6.8) |

Data are presented as numbers (%) and mean ± SD.

^*^Cardiac comorbidities included a history of heart failure, myocardial infarction, stable coronary disease, or coronary artery bypass graft surgery.

***Abbreviations:*** COPD, chronic obstructive pulmonary disease; FVC, forced vital capacity; FEV_1_, forced expiratory volume in 1 second; IgG, immunoglobulin G
