# Supplementary material for: The impact of IgG subclass deficiency on the risk of mortality in hospitalized patients with COPD
Source: Respir Res. 2022 May 31;23:141. doi: 10.1186/s12931-022-02052-3 (PMC9158163; doi:10.1186/s12931-022-02052-3)
Supplement: Supplementary file 4 — Additional file 4. Table S4. Unadjusted and adjusted HRs related to 1-year mortality according to IgG subclass deficiency in stable COPD patients. [file 12931_2022_2052_MOESM4_ESM.docx]

**Table S4.** Unadjusted and adjusted HRs related to 1-year mortality according to IgG subclass deficiency in stable COPD patients

| Type of IgG deficiency | Number at risk | 1-year mortality | Unadjusted model | | Adjusted model^*^ | |
| --- | --- | --- | --- | --- | --- | --- |
|  |  |  | Unadjusted HR  (95% CI) | p value | Adjusted HR  (95% CI) | p value |
| IgG1 deficiency | 1 | 0% (0/1) | NA^†^ | NA^†^ | NA^†^ | NA^†^ |
| IgG2 deficiency^†^ | 11 | 18.2% (2/11) | 2.50 (0.45–13.78) | 0.294 | NA^‡^ | NA^‡^ |
| IgG3 deficiency | 10 | 10% (1/10) | 1.68 (0.20–13.98) | 0.630 | 4.02 (0.32–51.13) | 0.284 |
| IgG4 deficiency | 15 | 13.3% (2/15) | 2.25 (0.46–11.17) | 0.319 | 2.14 (0.39–11.84) | 0.384 |

Data are presented as number, percentage, or ratios (95% CIs).

^*^Adjusted for age, sex, ethnicity (white vs. other ethnicities), smoking status (current vs. non-current), asthma status, and cardiac comorbidity status.

^†^No event of mortality.

^‡^The proportional hazards assumption was not met.

***Abbreviations:*** HR, hazard ratio; IgG, immunoglobulin G; COPD, chronic obstructive pulmonary disease; CI, confidence interval.
