# Supplementary material for: The impact of IgG subclass deficiency on the risk of mortality in hospitalized patients with COPD
Source: Respir Res. 2022 May 31;23:141. doi: 10.1186/s12931-022-02052-3 (PMC9158163; doi:10.1186/s12931-022-02052-3)
Supplement: Supplementary file 5 — Additional file 5. Table S5. Unadjusted and adjusted HRs related to 1-year mortality according to the number of IgG subclass deficiency in stable COPD patients. [file 12931_2022_2052_MOESM5_ESM.docx]

**Table S5.** Unadjusted and adjusted HRs related to 1-year mortality according to the number of IgG subclass deficiency in stable COPD patients

| Number of IgG deficiency | Number at risk | 1-year mortality | Unadjusted model | | Adjusted model^*^ | |
| --- | --- | --- | --- | --- | --- | --- |
|  |  |  | Unadjusted HR  (95% CI) | p value | Adjusted HR  (95% CI) | p value |
| 0 | 104 | 5.8% (6/104) | Reference |  | Reference |  |
| 1 | 20 | 5.0% (1/20) | 0.85 (0.10–7.02) | 0.876 | 0.77 (0.09–6.80) | 0.812 |
| 2 or more | 8 | 25.0% (2/8) | 4.24 (0.86–21.01) | 0.077 | 5.15 (0.83–31.76) | 0.078 |

Data are presented as number, percentage, or ratios (95% CIs).

^*^Adjusted for age, sex, ethnicity (white vs. other ethnicities), smoking status (current vs. non-current), asthma status, and cardiac comorbidity status.

***Abbreviations:*** HR, hazard ratio; IgG, immunoglobulin G; COPD, chronic obstructive pulmonary disease; CI, confidence interval.
